# ^18^F-FDG PET/CT identifies isolated metastasis of renal cancer in a patient with end-stage renal disease: A case report

**DOI:** 10.1097/MD.0000000000043595

**Published:** 2025-07-25

**Authors:** Chongling Duan, Lin An

**Affiliations:** a Department of PET/CT, Jining NO.1 People’s Hospital, Shandong First Medical University, Jining, Shandong Province, China.

**Keywords:** end-stage renal disease, PET/CT, renal cancer

## Abstract

**Rationale:**

Individuals with end-stage renal disease have a considerably higher rate of malignant tumors, especially renal cancer, in comparison to the general population. However, cases in which bone metastases in the humerus are the initial clinical presentation that results in a diagnosis of renal cancer are extremely rare. Diagnosing renal cancer in patients with end-stage renal disease can be challenging when the clinical symptoms are atypical and do not present the “renal cancer triad.” Our case report highlights the diagnostic importance of positron emission tomography/computed tomography (CT) imaging, increases clinicians’ awareness of the disease, explores the potential etiology of renal cancer associated with end-stage renal disease, and provides insights into diagnostic and therapeutic strategies.

**Patient concerns:**

A 58-year-old man, who has undergone multiple renal transplants and maintenance dialysis therapy for over 20 years due to renal failure, arrived at our hospital with complaints of left shoulder pain and progressive worsening. CT revealed unexplained osteolytic destruction and fracture in the left humerus. The ^18^F-fluorodeoxyglucose positron emission tomography/CT scan detected soft tissue lesions in the left kidney and exhibited increased fluorodeoxyglucose uptake.

**Diagnoses:**

Upon conducting a biopsy on the patient’s left humerus, metastatic renal cancer was diagnosed.

**Interventions:**

The patient selected conservative treatment, and the injured humerus was immobilized to stabilize the affected area. There was no additional active treatment for renal cancer.

**Outcomes:**

As of the time of submission, the patient’s pain had markedly intensified, requiring oral pain medication for symptomatic relief.

**Lessons:**

It is indeed rare for bone metastasis in the humerus to be the initial clinical sign that leads to the diagnosis of renal cancer in patients with end-stage renal disease. Through this case report, we aimed to enhance awareness and deepen understanding of renal cancer associated with end-stage renal disease.

## 1. Introduction

End-stage renal disease (ESRD) has an estimated prevalence of 4 to 7 million cases, and this number is on the rise.^[1]^ Patients suffering from ESRD have a 50-fold increased risk of developing renal cancer compared to those with normal renal function.^[2]^ Metastases to the bones from renal cancer (29.5%) were the second most prevalent site, just behind lung metastases (45.2%). However, in bone metastases, the most common sites were the axial skeleton, such as the pelvis (48%), ribs (48%), and vertebrae (42%). Meanwhile, renal cancer often leads to multiple bone metastases, with isolated cases being exceedingly rare, affecting only 1.4% to 2.5% of patients.^[3]^ Earlier studies have indicated that ESRD-associated renal cancer is likely to possess advantageous clinical, pathological, and prognostic traits, frequently presenting at an earlier stage and with a lesser number of metastases.^[4]^ The early indicators of individuals with ESRD-associated renal cancer do not include the classic triad of renal cancer symptoms (abdominal pain, abdominal mass, hematuria), making it challenging to identify these patients.^[5]^ We describe a case of ESRD-associated renal cancer that initially presented with isolated bone metastasis in a humerus. Our detailed description of the onset and progression of ESRD-associated renal cancer aims to increase and broaden the current awareness of this disease, thereby facilitating earlier diagnosis and the development of appropriate treatment options.

## 2. Case description

**Figure 1.**
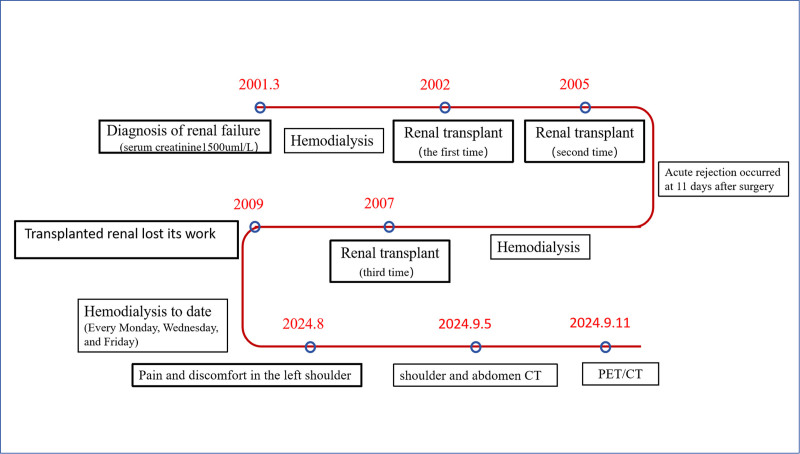
The timeline depicts the patient’s medical history and the course of diagnosis and treatment.

**Figure 2.**
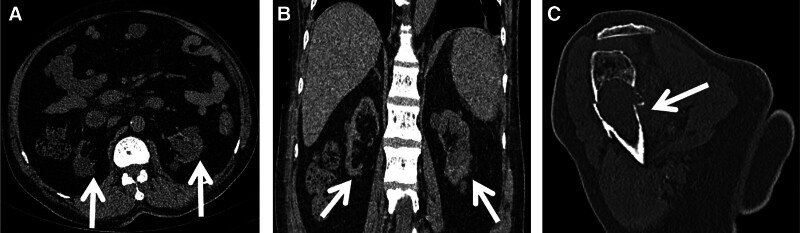
A 58-year-old patient with end-stage renal disease (ESRD) experiencing discomfort and pain in the left shoulder for the past month. CT scans in the axial (A) and coronal (B) planes from a year ago depict numerous cysts (>3). Recent coronal (C) CT imaging indicates bone destruction and a fracture in the proximal left humerus. CT = computed tomography.

For the purpose of clearly delineating the patient’s historical medical background and the sequence of diagnostic and therapeutic interventions, we have elected to represent these details on a chronological timeline (Fig. 1). An individual, a 58-year-old male, is diagnosed with ESRD. His medical history includes renal failure that has persisted for over 2 decades, during which he has received 3 renal transplants. Since 2009, when his transplant kidney ceased to function, he has been on a routine dialysis program. In the year 2012, the medical operation known as “total parathyroid gland resection with right upper arm transplantation” was executed. With the extension of the patient’s dialysis duration, the kidneys began to develop numerous cysts (>3). As shown by Figure 2A and B depicted the abdominal computed tomography (CT) scan from July 14, 2023. In August 2024, the patient began to experience discomfort and pain in the left shoulder, which was particularly noticeable during activities that required lifting the arm upwards. A CT scan revealed unexplained bone destruction in the left humerus, accompanied by a fracture (Fig. 2C).

After admission, the patient underwent further examinations, including hematological tests, tumor marker assessments, and plain CT scans. The tumor markers CEA and SCC were found to be slightly elevated at 5.32 ng/mL (normal range: 0–5 ng/mL) and 2.2 ng/mL (normal range: 0–1.5 ng/mL), respectively. In addition, no irregularities were detected in AFP, CA199, CA724, PSA, and Cyf211. The results of the hematological analysis did not display any clear signs of abnormalities. A plain CT scan of the abdomen disclosed several renal cysts, and no considerable tumor lesions were found.

**Figure 3.**
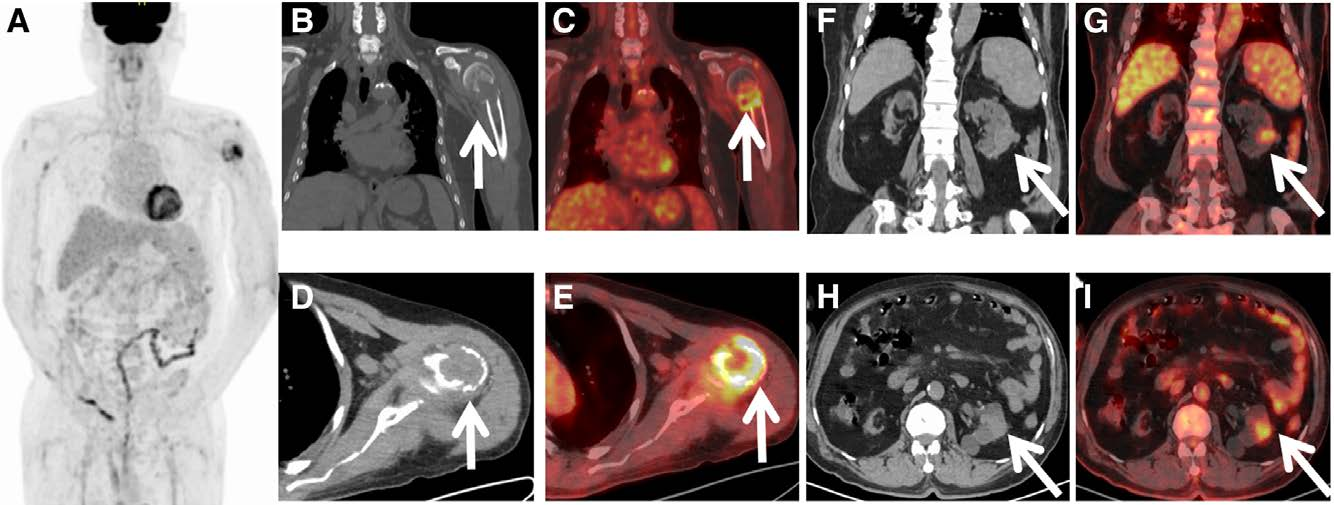
The patient underwent an ^18^F-FDG PET/CT scan based on suspected malignant tumor. The MIP (A) images show a high ^18^F-FDG uptake in the left humerus. Coronal (B) and axial (D) CT images show bone destruction and soft tissue formation in the humerus, with fractures (arrows). The PET/CT fusion images (C, E) show high uptake of ^18^F-FDG (SUVmax, 8.6). Coronal (F) and axial (H) CT images show a soft tissue lesion in the lower part of the left kidney. PET/CT fusion images (G, I) show moderate uptake of ^18^F-FDG (SUVmax, 3.1). ^18^F-FDG PET/CT = ^18^F-fluorodeoxyglucose positron emission tomography/computed tomography, CT = computed tomography, MIP = maximum intensity projection.

Considering the patient’s inability to exclude cancer as a likely diagnosis, a detailed PET/CT tumor screening was carried out (Fig. 3). Maximum intensity projection (MIP) image showed higher fluorodeoxyglucose (FDG) uptake in the left humerus. The left humerus revealed bone destruction and soft tissue formation, along with a high uptake of ^18^F-FDG (SUVmax, 8.6). Additionally, localized moderate FDG uptake was observed in the left renal, and the CT scan revealed a mass about 23 × 20 mm. Moreover, there was no significant tumor-related FDG uptake in the residual PET/CT field, and just a isolated lesion was detected within the entire skeletal system. Based on these findings, PET/CT initially diagnosed renal occupation with isolated humeral metastasis. Nonetheless, with the rarity of this disease presentation, further humeral pathology was warranted, and the biopsy pathology confirmed metastatic renal clear cell cancer. The immunohistochemistry results were PAX8 (+), CA9 (+), TFE3(−), PTH(−), Ki67(+60%), SOX10 (−), CD163 (−). Regrettably, the patient decided on conservative treatment, the humerus was immobilized, and the affected area was stable, with no additional active treatment for renal cancer being undertaken.

## 3. Discussion

With the growing number of patients suffering from ESRD worldwide, there has been a substantial rise in the frequency of associated cancers, with renal cancer being the most prevalent.^[6]^ The development of renal cancers in patients with ESRD is intricate, with no singular cause having been pinpointed. Nevertheless, research has brought to light several mechanisms that could play a role in the development of renal cancers in patients with ESRD. Acquired cystic renal disease has been the primary hypothesis explaining the epidemiology of renal cancer in patients with ESRD. Acquired cystic renal disease was first described by Dunnil et al in 1977, and it is characterized by the presence of 3 or more cysts in each renal in patients undergoing dialysis.^[7]^ Within the initial 3-year period of dialysis treatment, it is estimated that 10–20% of patients will develop acquired cystic renal disease. However, after 10 years of dialysis, over 90% of patients exhibit acquired cystic renal disease.^[8]^ Studies suggest that the uncontrolled cell growth and proliferation within hyperplastic cystic tissues are the main factors contributing to tumor development and progression.^[9]^ Renal cysts, renal cancer, and dialysis are closely related. The risk of renal cancer increases with the prolonged duration of dialysis in patients.^[10]^ Extended dialysis treatment, spanning over 10 years, is positively linked to an elevated risk of renal cancer.^[11]^ In this case, the patient had a medical history of over a decade on dialysis and experienced multiple cysts in both kidneys, which ultimately resulted in the onset of renal cancer. Transplantation complicates the intricate, bidirectional causal relationship between renal cancer and ESRD. After a transplant, the use of numerous immunosuppressive drugs is necessary, which can somewhat raise the risk of renal tumors. Patients with weakened immune systems are susceptible to suppressed defense responses and the unchecked growth of oncogenic viruses due to impaired immune monitoring in their tissues.^[12]^ Additionally, the category of immunosuppressive drugs could influence the magnitude of the elevated risk for tumors.^[13,14]^ Furthermore, the heightened levels of oxidative stress, the upsurge in antioxidant proteins, peroxiredoxin, and thioredoxin, are regarded as potential factors contributing to the increased prevalence of renal cancer in ESRD patients compared to those with normal renal function.^[15]^

Improved dialysis techniques have prolonged the life expectancy of individuals suffering from ESRD, and the early identification of cancer is expected to be a significant predictor of outcomes. Tumor markers are not reliable in patients with renal replacement therapy, as they exhibit a high rate of false positives or low sensitivity. Special attention is required when interpreting renal excreted tumor markers, as they are large molecules that are not easily filtered out by dialysis, potentially causing incorrect experimental results. Markers such as CA125, CEA, SCC, and NSE are particularly prone to this issue.^[16]^ Conducting clinical imaging screenings on dialysis patients to detect renal cancers in their early stages would be extremely advantageous.^[17]^ It should be noted that the effectiveness of CT, MRI, and US in identifying renal cancer in kidneys with multiple cysts is frequently questionable. This is due not only to the high number of cysts found in the kidneys of these patients, but also because benign cysts have a tendency to bleed or become infected. Regarding the enhanced imaging, the detection of renal tumors can be challenging due to the presence of numerous cysts, especially when the enhancement is ambiguous or only subtle.^[18]^ Fortunately, investigations have indicated that the renal uptake of FDG is diminished in patients with severely impaired renal function, which enhances the detection of renal malignancies in FDG PET/CT imaging.^[17,19]^ The authors demonstrated the effectiveness of FDG PET/CT in assessing renal cancer in patients on dialysis, as revealed in a cross-sectional study of FDG PET/CT scans of the urinary tract in 150 dialysis patients.^[17]^ These findings suggest that FDG PET/CT plays a crucial role in the diagnosis of renal cancer in patients with ESRD. Here, an unusual humeral metastasis was the initial clinical presentation, showcasing the advantage of a wide field of view and high sensitivity for PET/CT imaging, and the final results also indicate that FDG PET/CT can detect renal cancer in patients with ESRD. Another research has explored the efficiency of choline PET in diagnosing renal cancer, revealing that its uptake is more evident than that of ^18^F-FDG PET/CT.^[20]^ However, it is less readily accessible than FDG which makes it a less appropriate screening tool. In conclusion, the authors are convinced that ^18^F-FDG PET/CT is beneficial for tumor screening in patients with ESRD, especially for renal cancers, and thus conducive to the formulation of the next treatment plan.

For localized renal cancer (stages I–III), surgical removal of the tumor is the preferred therapy and is correlated with a good prognosis.^[21]^ Currently, there is no established treatment standard or guideline for renal cancer with bone metastasis. The median overall survival time after the occurrence of bone metastasis in renal cancer spans between 12 and 28 months.^[22]^ Usually, bone metastasis treatment in renal cancer is mostly a palliative treatment, including radiotherapy, chemotherapy, immunotherapy, targeted therapy, bisphosphonates, and analgesics.^[23]^ Surgical resection of bone metastases has been reported to improve the prognosis of patients, especially in cases of isolated metastases.^[24]^ During the years leading up to the 2000s, cytokine therapy was the main treatment for patients with advanced renal cancer, such as interleukin-2 (IL-2) and interferon alpha (IFN-α). In recent years, immune checkpoint inhibitors (ICIs) and tyrosine kinase inhibitors (TKIs) are often considered as the first-line treatment for advanced renal cancer. ICIs are large biological immune-therapy molecules that can be either complete antibodies or antigen-binding fragments (Fab), which induce cell death pathways in tumor cells by promoting the activation of T-cells. It’s unfortunate that administering ICIs to renal cancer patients undergoing renal transplantation for ESRD has a detrimental effect on graft survival by elevating the incidence of rejection. TKIs are designed to target and affect the activity of specific tyrosine kinase proteins. The fundamental mechanism of TKIs in renal cancer entails targeting diverse subtypes of vascular endothelial growth factor (VEGF) receptors, thereby inhibiting tumor angiogenesis. It is worth noting that TKIs had no associated toxicity when compared to conventional cytokine therapy.

There is a lack of consensus in the literature regarding the prognostic differences between renal cancer patients with and without ESRD. According to Neuzillet et al renal cancer patients suffering from ESRD tend to have better survival outcomes than those without ESRD.^[4]^ However, the study conducted by Hayami and colleagues reveals a contrary outcomes.^[25]^ Research has indicated that extended dialysis duration, larger tumor size, advanced pathological stage, grade 4 tumors, lymphovascular invasion, and the existence of a sarcomatoid components are key factors that impact patient prognosis. Longer periods of dialysis (HR 2.11; 95% CI: 1.4–4.84) and the presence of lymphovascular invasion (HR 29.55; 95% CI: 1.21–387.88) were identified as independent predictors.^[26]^

## 4. Conclusion

In summary, this study presented a distinctive case of renal cancer in the context of ESRD, where the initial symptom was isolated bone metastasis in the humerus. Emphasizing the distinctive benefits of ^18^F-FDG PET/CT in diagnosing tumors in patients with ESRD. The main purpose of this paper is to raise clinicians’ awareness of ESRD-associated renal cancer, and to decrease the incidence of missed and incorrect diagnoses, thereby facilitating timely and effective treatment. Further research is needed to better understand the treatment and prognosis of these patients, which will require more cases involving active intervention and long-term follow-up.

## Author contributions

**Conceptualization:** Chongling Duan.

**Supervision:** Lin An.
